# Giants

**Published:** 1891-07

**Authors:** 


### GIANTS.

The existence of whole nations of gigantic persons may well be questioned; but there can be no reasonable doubt of the reality of certain individuals, whose height has greatly exceeded that of men in general. The giant as a curiosity has been seen by nearly every boy in the museum and in the circus. The stories of great giants of the past ages are more than interesting. The exact height of Og, the King of Bashan, has been frequently given; some supposing him to have been more than twelve feet in height, while others think his stature did not exceed eleven feet. In like manner the giant Goliath of the Bible, is generally computed to have been nine feet nine inches high; but commentators suppose that he might have been fully eleven feet.

The Emperor Maximinius was nine feet in height, and several other Romans of equal stature are said to have lived during the reign of Augustus. Accounts are contained in the philosophical transactions of the Royal Society, of human skeletons dug up in England, measuring eight and nine feet in length. These were probably the remains of Romans. Many fabulous and contradictory stories have been told at one time of the Patagonians who, according to travellers, have been a race of giants. An English official once declared that he had measured the bones of men, in sepulchres in South America, between eleven and twelve feet high. Turner, the naturalist, declares that he once saw, upon the coast of Brazil, a race of gigantic savages, one of whom was twelve feet in height. The declaration of Turner is all the more credible, by the statement of M. Thevet, of France, who, in his description of

America, published in Paris, in 1575, asserts that he saw and measured the skeleton of a South American which was eleven feet five inches in length. To these remarkable instances may be added a well proportioned living man, whom Diemerbrock saw at Utrecht, measuring eight feet six inches. Dr. Becamus, an ancient scholar, reports having seen a youth of nine feet, a man of ten feet and a woman nine feet in height.

Walter Parsons, who acted as porter to King James I, of England, was seven feet six inches in stature. But the Chinese claim to have had men among them in the last century who were fifteen feet high. However, this report may have no more foundation than the chronological fables of the sons of China.

